# Long non‐coding RNA MALAT1 regulates angiogenesis following oxygen‐glucose deprivation/reoxygenation

**DOI:** 10.1111/jcmm.14204

**Published:** 2019-02-19

**Authors:** Chengya Wang, Youyang Qu, Rui Suo, Yulan Zhu

**Affiliations:** ^1^ Department of Neurology Second Affiliated Hospital of Harbin Medical University Harbin China

**Keywords:** angiogenesis, endothelial cells, ischaemia/reperfusion, MALAT1, oxygen‐glucose deprivation/reoxygenation

## Abstract

Long non‐coding RNAs (lncRNAs) have been identified as playing critical roles in multiple diseases. However, little is known regarding their roles and mechanisms in post‐stroke angiogenesis. Our studies focused on deciphering the functional roles and the underlying mechanisms of the lncRNA metastasis‐associated lung adenocarcinoma transcript 1 (MALAT1) in the process of angiogenesis following oxygen‐glucose deprivation/reoxygenation (OGD/R). We characterized the up‐regulation of MALAT1 expression in the process of angiogenesis after hypoxic injury in vivo and in vitro. We further showed that compared with the empty vector, MALAT1 knockdown had significantly reduced the capacity for angiogenesis, which was measured by 3‐(4,5‐dimethylthiazol‐2‐yl)‐2,5‐diphenyltetrazolium (MTT), scratching, cell cycle and immunofluorescent staining. Thus, our findings suggest that MALAT1 may mediate proangiogenic function in OGD/R. To further explore the potential mechanisms, we used lentiviruses expressing shMALAT1 and empty vector; the results revealed that shMALAT1 reduced the expression of 15‐lipoxygenase 1 (15‐LOX1), vascular endothelial growth factor (VEGF) and the phosphorylation of signal transducers and activators of transcription 3 (pSTAT3). Taken together, our results are the first to propose that MALAT1 may regulate angiogenesis through the 15‐LOX1/STAT3 signalling pathway, and they may provide a critical target for the treatment of hypoxic injury and an avenue for therapeutic angiogenesis.

## 1 INTRODUCTION

Stroke has been shown to be one of the leading causes of mortality and the most common cause of disability in adults in most countries.^1^ Although clinically effective drug (recombinant tissue plasminogen activator, rt‐PA) therapy has been achieved for acute ischaemic stroke, the majority of patients miss the optimal opportunity for vascular recanalization, owing to the narrow therapeutic window and safety concerns.^2^ Thus, there remains an urgent need to decipher the potential mechanisms of stroke, understanding those mechanisms will be beneficial for the clinical therapy of patients.

A novel class of non‐coding RNAs over 200 nucleotides in length, known as long non‐coding RNAs (lncRNAs), is pervasively transcribed in the mammalian genome.^3^ Within the past decade, aberrantly expressed lncRNAs have been demonstrated to act as key regulators of post‐transcriptional gene expression in pathological aspects of ischaemic stroke.^4^ However, less is known about the participation of lncRNAs in regulatory mechanisms of angiogenesis following cerebral I/R. MALAT1 is a highly abundant and evolutionary conserved lncRNA that was first described as being associated with the metastasis of lung tumours.^5^ Recent studies have shown that MALAT1 is closely associated with endothelial cell repair after ischaemia.^6^, ^7^ Furthermore, in a remarkable discovery by Zhang et al., RNA‐sequencing revealed that MALAT1 is significantly up‐regulated in oxygen‐glucose deprivation (OGD)‐responsive endothelial cells.^8^ However, the biological function and molecular mechanisms of action of MALAT1 in angiogenesis following I/R have not been previously reported. Therefore, we will evaluate whether MALAT1 is involved in angiogenesis after cerebral I/R and if yes, what is the detail mechanism.

## 2 MATERIALS AND METHODS

**Figure 1:**
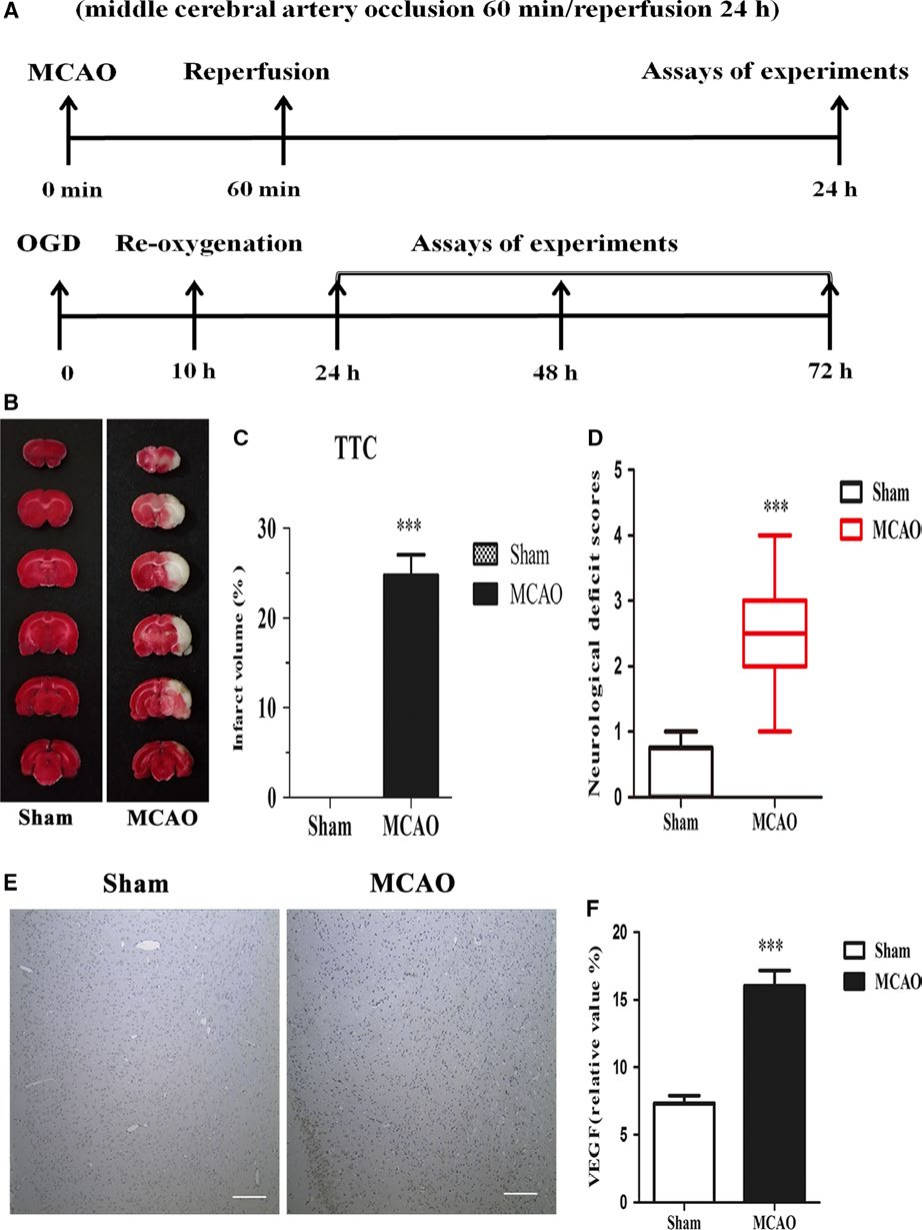
Summary of experimental protocols, ischaemic infarct volume and immunohistochemical results. (A) Study design and timeline of experiments in vivo and vitro. (B) Representative triphenyltetrazolium chloride staining results from the sham and MCAO groups. (C) The hemispheric infarct ratios in comparable sections from two groups (n = 8/group). (D) Neurological deficit scores. (E) Representative vascular endothelial growth factor (VEGF) immunohistochemistry, scale bar = 50 μm. (F) Statistical analysis of the mean density of VEGF (n = 7/group). Data are presented as the mean ± SEM. ***P < 0.001 vs sham group

Male C57BL/6J mice aged 10‐12 weeks and weighing 25‐30 g were obtained from the Animal Center of the Second Affiliated Hospital of Harbin Medical University used in the present study. All of the procedures and ethical considerations were approved by the Committee for Experimental Animal Use and Care of the Second Affiliated Hospital of Harbin Medical University, China. All procedures followed the guidelines of the Administration of Laboratory Animals and Humane Treatment of Laboratory Animals in China. Moreover, the experiments with animals were performed in compliance with the ARRIVE (Animal Research: Reporting In Vivo Experiments) guidelines. Furthermore, the study was divided into vivo and vitro portions. The detailed experimental protocol is shown in Figure 1A.

### 2.1 Focal cerebral ischaemia

Mice were anaesthetized with 1.5% isoflurane in 30% oxygen with a face mask. After a midline to the right skin incision, the right common carotid artery and the internal carotid artery were exposed and the branches of the common carotid artery were electrocoagulated. A 2‐cm length of 7‐0 rounded silicone‐rubber‐coated monofilament (L3600, Jialing, Guangzhou, China) was introduced from the common carotid artery up to internal carotid artery until regional cerebral blood flow (rCBF) reduction (>70%).^9^ After 60 minutes, the filament was removed to allow reperfusion for 24 hours. Sham group animals were subjected to similar operations to expose the internal carotid artery but without the occlusion of the middle cerebral artery. Subsequently, the Zea Longa score was used to determine the degree of neurological deficit.^10^ Zea Longa scores are divided into 5‐grade, as shown in Table 1.

**Table 1:** The criteria of Zea Longa Score

Score	Neurologic impairment	Grade
0	No obvious neurologic impairment	Normal
1	Failure to extend the right forepaw	Mild
2	Circling to the right	Moderate
3	Falling to the right	Serious
4	Mice can't walk spontaneously	Unsuccessfully

### 2.2 Measurement of cerebral infarction volume

Mice were given an overdose of anaesthesia with isoflurane and decapitated (n = 8/group) 24 hours after reperfusion. The brains were carefully removed, and 2‐mm‐thick coronal sections were collected in 1% 2,3,5‐triphenyltetrazolium chloride (TTC, Sigma, USA) for 15 minutes at 37°C, followed by overnight fixation in 4% paraformaldehyde (pH 7.4) (Solarbio, Beijing, China).^11^ Sections were imaged and digitized. Then, the images were analysed with ImageJ software to determine the infarct area. Infarct size was expressed as a percentage of hemispheric area.^12^

### 2.3 Immunohistochemical analysis

Histological sections (4 μm in thickness) prepared from paraffin‐embedded tissue samples of ischaemic brains were used for the immunohistochemical analysis. Sections were blocked using 0.3% H_2_O_2_ for 10 minutes. Subsequently, the sections were incubated with anti‐VEGF primary antibody (1:100, ab1316, Abcam, Cambridge, MA, USA) for 2 hours at room temperature. The brain tissues were then rinsed with phosphate‐buffered saline (PBS). Next, the sections were incubated with biotinylated goat anti‐mouse IgG secondary antibody (Zsbio, Beijing, China) for 30 minutes. 3,3‐Diaminobenzidine (DAB) was used as the chromogen. No primary antibody was used in the secondary only control. After staining, the mean density of VEGF was evaluated in three randomly selected fields per section under a microscope (Nikon, Tokyo, Japan).

### 2.4 Fluorescence in situ hybridization (FISH)

The techniques and labelled single‐stranded RNA probes used for in situ hybridization were used as previously described.^13^ The expression of MALAT1 was determined according to the instructions of the fluorescence in situ hybridization (FISH) kit used in our studies (Boster, Wuhan, China). Hybridization was performed on cryostat‐cut coronal brain sections (10 μm) by incubating with a labelled RNA probe overnight at 48°C. The sections were washed, dehydrated, coated, developed and exposure.

### 2.5 Cell cultures

A mouse brain microvascular endothelial cell line was purchased from Fuheng (Shanghai, China) and it was certified by Biosystems. The cells were incubated in six‐well plates (Corning, New York, USA). The culture medium was Dulbecco's modified Eagle's medium (DMEM, Corning, New York, USA) supplemented with 10% foetal bovine serum (FBS, CellMax, Beijing, China), 100 U/mL penicillin and 100 μg/mL streptomycin. The cell cultures were maintained in a cell incubator at 37°C and 5% CO_2_.^14^

### 2.6 OGD/R model of ECs

Ischaemia was mimicked in vitro as follows: first, cells were washed with PBS (Solarbio, Beijing, China). Next, the cells were cultured in glucose‐free DMEM (GIBCO, New York, USA) without FBS in a hypoxia incubator (95% N_2_, 5% CO_2_) at 37°C for 10 hours. After OGD exposure, the medium was replaced with absolute medium containing 10% FBS and cultured under normoxic conditions (95% air, 5% CO_2_, 21% FiO_2_) to perform OGD/R model. Cells were incubated at 37°C in normal‐glucose DMEM under normoxic conditions as a control. Importantly, the hypoxic chamber was previously sealed and placed in the 37°C thermostat container (SPX‐150C, Boxun, Shanghai, China), where it was flushed in advance with a gas mixture of 95% N_2_, 5% CO_2_ for 30 min at the rate of 2 L/min. After flushing, the concentration of O_2_ was managed with a gas monitor (Smart Sensor, Hong Kong, China). The concentration of O_2_ was maintained at less than 1%.

### 2.7 Cell viability assay

MTT (Beyotime, Beijing, China) was used to evaluate cellular viability according to the manufacturer's instructions. Cells at a seeding rate of per 2 × 10^4^ well were incubated in 96‐well plates and added to culture medium with 10 μL of 5 mg/mL MTT reagent at 37°C for 4 hours under normal growth conditions. Then, the cells were lysed at room temperature by adding 100 μL of dimethyl sulfoxide (DMSO, Solarbio, Beijing, China) for 10 minutes. Finally, cell viability was determined by measuring the optical density (OD) at 490 nm in a universal enzyme marker (Bio‐Rad, Shimadzu, Japan). Percent change relative to the control was calculated as a measure of cell viability.

### 2.8 Immunofluorescent staining analysis

Cells were cultured on coverslips in 24‐well plates and then fixed with 4% paraformaldehyde for 20 minutes at room temperature. Subsequently, the cells were permeabilized with 1% TritionX‐100 (Solarbio, Beijing, China) solution for 20 minutes. Next, goat serum was added to block non‐specific binding sites at room temperature for 60 minutes. Anti‐CD31 primary antibody (1:100, ab222783, Abcam, Cambridge, MA, USA) was added to the cells and incubated at 4°C overnight. The following day, each section was washed with PBST (PBS with 0.1% Tween‐20) and incubated with Cy3 goat anti‐rabbit IgG (H + L) secondary antibody (1:100, ABclonal, Boston, USA) at 37°C for 2 hours. Anti‐fade DAPI (Beyotime, Shanghai, China) solution was needed. Immunofluorescent staining was observed under a laser confocal microscope (Nikon, Tokyo, Japan). Finally, the CD31‐positive cells were counted using ImageJ software, and the mean total number of positive cells was considered the degree of cerebral angiogenesis induced by ischaemic stroke.^15^

### 2.9 Cell migration

Migration was assessed using a scratching assay. A six‐well plate was divided with a marker pen approximately every 0.5‐1.0 cm. A total of 5 × 10^5^ cells per well were incubated in a six‐well plate and grown to confluence overnight. In this confluent monolayer, a single scarification was made with 200‐μL plastic pipette tip. The healing of the scratch was visualized at the indicated time (48 hours) using a microscope (×4). The area of the scratch was analysed using ImageJ software.

### 2.10 Quantitative real‐time polymerase chain reaction

Total RNA was extracted from I/R cerebral tissue or OGD/R cells using TRIzol Reagent (Invitrogen, California, USA). Reverse transcription was carried out with a cDNA Reverse Transcription kit (Roche, Indianapolis, USA). Subsequently, the product from reverse transcription was amplified with FastStart Universal SYBR Green Master (Roche, Indianapolis, USA) in a 10‐μL reaction volume using a Bio‐Rad CFX96 Detection System (Bio‐Rad, Shimadzu, Japan). Each sample was run in triplicate, and analysis of relative gene expression level was performed with β‐actin as the reference using the 2^−ΔΔCT^ method. The sequences of the specific primers used in our experiment are shown in Table Table 2.

**Table Table 2:** All of primer sequences in our experiment

Gene Names	primer (5′‐>3′)	Length of production	Gene ID
15‐LOX1 F	5′‐TGAACAGCTTGGTCGGTCTTG‐3′	221BP	NM_009660.3
15‐LOX1 R	5′‐ GTGATGAGCACAGGTGGAGGA‐3′		
VEGF F	5′‐ GTCCTCTCCTTACCCCACCTCCT‐3′	106BP	NM_001110267.1
VEGF R	5′& CTCACACACACAGCCAAGTCTCCT&3′		
MALAT1 F	5′&GAGTGTGTGATGTGAGACCTTG&3′	191BP	NR_002847.3
MALAT1 R	5′‐GCATTCTAATAGCAGCAGATTGG‐3′		
β‐actin F	5′‐ GGGAAATCGTGCGTGAC‐3′	176BP	NM_007393.5
β‐actin R	5′‐ AGGCTGGAAAAGAGCCT ‐3′		

### 2.11 Cell transfection

To generate a lentivirus expressing shMALAT1 and an empty vector, we cultured the cells in 6‐well plates for 12 hours, the culture medium was centrifuged to collect the lentivirus. A volume of 300 μL of each virus supernatant was added into 700 μL of fresh culture with 1 μL of polybrene (final concentration degree 8 ng/mL). The premixed viral infection was added to a new culture dish containing cells at no more 60% confluence. The culture medium was changed the following day. After 48 hours, the cells were screened with puromycin (Thermo Fisher, Shanghai, China) and then cultured for another 2 days. When the cells were harvested, some were frozen and the rest were used for the subsequent experiments.

### 2.12 Enzyme‐linked immunosorbent assay (ELISA)

The concentration of 15‐HETE was evaluated using an ELISA according to the manufacturer's instruction, as described previously.^16^ In order to measure the concentration of 15‐HETE after inhibition, the 15‐HETE kit (SinoBest, Shanghai, China) was employed. The calibration standards are assayed at the same time as the sample and the operator was allowed to produce a standard curve of OD vs 15‐HETE concentration. The concentration of 15‐HETE in the samples is then determined by comparing the OD of the samples to the standard curve.

### 2.13 Western blot analysis

Samples from cerebral tissues and cells were homogenized in lysis buffers (Solarbio, Beijing, China) with a phosphorylation inhibitor (Roche, Indianapolis, USA), and total protein was isolated as described previously.^17^ The total protein concentration for each sample was measured with a bicinchoninic acid (BCA) kit (Beyotime, Shanghai, China). Equal amounts of protein samples were subjected to SDS‐polyacrylamide gel electrophoresis (SDS‐PAGE), and the resolved protein bands were transferred onto a polyvinylidene fluoride (PVDF, Thermo Fisher, Shanghai, China) membrane. The PVDF membrane was blocked in TBS‐Tween 20 with 5% (w/v) non‐fat powdered milk for 60 minutes at room temperature. Target proteins were detected through incubation overnight at 4°C with primary antibodies against 15‐LOX1 (1:900; ab150051, Abcam, Cambridge, MA, USA), VEGF(1:1000; ab1316, Abcam, Cambridge, MA, USA), pSTAT3 (1:20000, ab76315, Abcam, Cambridge, MA, USA), STAT3 (1:1000‐2000, ab68153, Abcam, Cambridge, MA, USA,), and β‐actin (1:1000; Zsbio, Beijing, China) as the control. The membranes were then washed and incubated for 1 h with goat anti‐rabbit IgG and goat anti‐mouse IgG (diluted at 1:5000, ABclone, Boston, USA). Finally, the antigen‐antibody complexes were detected using an enhanced chemiluminescence (ECL) reagent kit (Vazyme, Nanjing, China). The intensity of each band area was quantified using ImageJ.

### 2.14 Statistical analysis

Data are expressed as the mean ± standard error (SE), except for the neurological deficit scores, which are presented as the medians and interquartile ranges. Student's *t* test or two‐way ANOVA was used for all pairwise comparisons. *P *< 0.05 indicated statistical significance. The statistical analyses were performed using GraphPad Prism version 5.5 for Windows (GraphPad Software, San Diego, CA, USA).

## 3 RESULTS

### 3.1 Angiogenesis is activated after ischaemic injury

Sixty C57BL/6J mice were subjected to middle cerebral artery occlusion MCAO 1 hour before reperfusion for 24 hours; six mice died and the other fifty‐four mice survived. We evaluated the ischaemic infarct volumes, and mice were randomly selected for TTC staining after surgery. Infarct volume was significantly increased in the MCAO group compared with the sham group (Figure 1B). The neurological deficit scores were estimated in the sham and MCAO groups (Figure 1C). Furthermore, we employed VEGF immunohistochemistry to analyse the amount of microvasculature in the ischaemic penumbra 24 hours following cerebral ischaemic stroke. The results demonstrated that VEGF was up‐regulated in the MCAO group compared with sham group (Figure 1D), indicating that angiogenesis is activated upon ischaemic injury.

### 3.2 OGD/R promotes ECs proliferation, migration and CD31 positive cell expression

**Figure 2:**
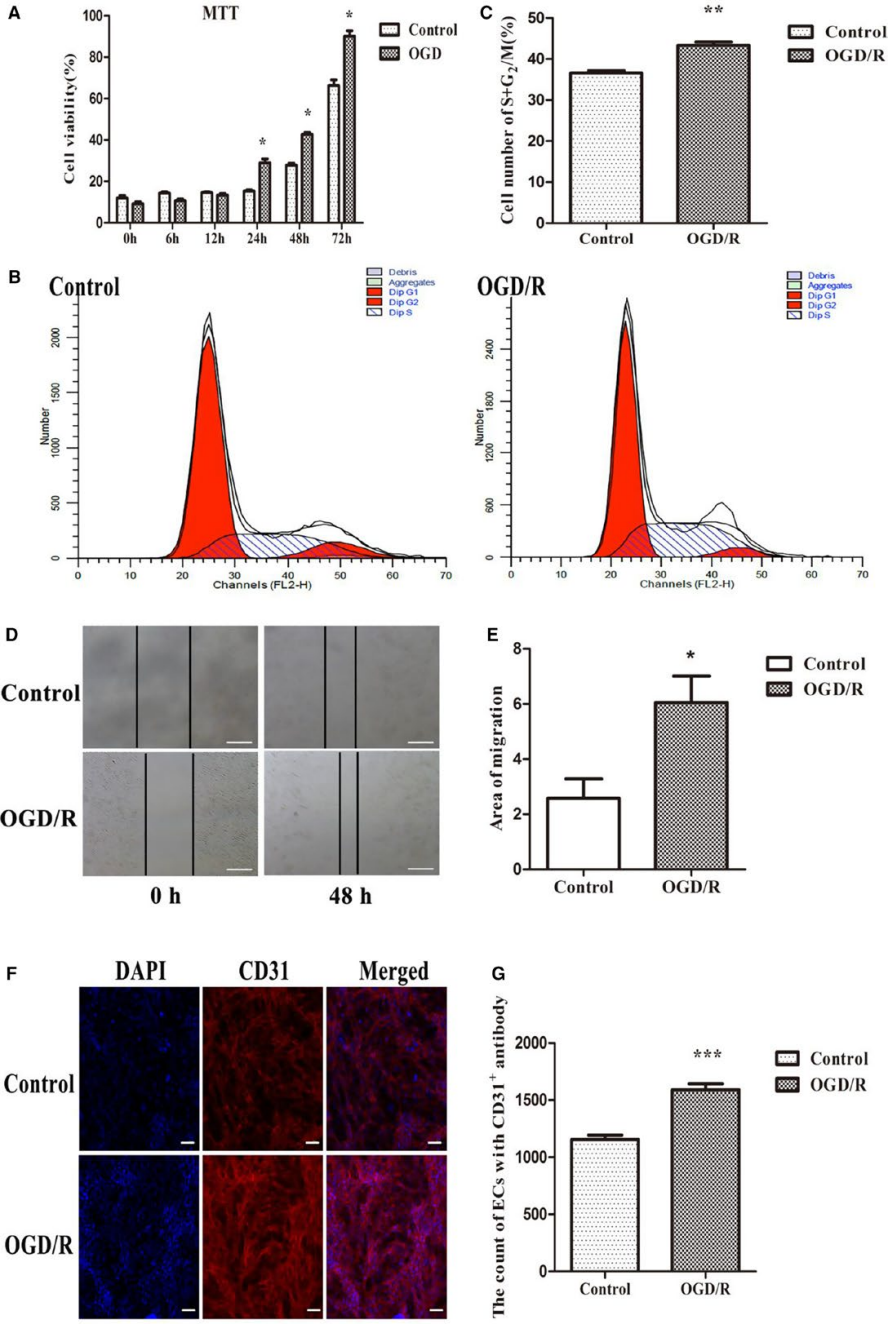
The survival rate, proliferation and migration of brain microvascular endothelial cells between the oxygen‐glucose deprivation/reoxygenation (OGD/R) and control groups. (A) Time‐dependent increase of cell viability in the OGD/R group compared with the control group. (B) Representative cell cycle distribution in comparable samples from the control and OGD/R groups. (C) Statistical analysis of the number of S + G_2_/M cells from two groups (n = 6/group). (D) Scratch‐wounding assay in OGD/R compared with control endothelial cells. (E) Statistical analysis of the migration capacity between the OGD/R and control groups (n = 6/group). (F) Representative immunofluorescent staining of the control and OGD/R groups, scale bar = 50 μm. (G) Statistical analysis comparing angiogenesis between the control and OGD/R groups (n = 6/group). Data are presented as the mean ± SEM. *P < 0.05 vs control, **P < 0.01 vs control, ***P < 0.001 vs control

Brain microvascular endothelial cells were cultured to confluence, and then subjected to OGD for 10 hours. Next, they were cultured under normoxic conditions for different lengths of time, including 0, 6, 12, 24, 48 and 72 hours. MTT and representative cell cycle stages were used to measure the cell viability and proliferation. A scratching assay and immunofluorescent staining were used to measure the migration capacity and number of endothelial cells following OGD/R. In our studies, the results obtained following OGD/R treatment revealed that a hypoxic stimulus could significantly promote endothelial cell survival depending on the increase in reoxygenation time (Figure 2A). Consequently, we selected reoxygenation for 48 h for subsequent assays. Accordingly, the functional analysis associated with angiogenesis of ECs following OGD/R, including the cell proliferation assay (Figure 2B,C), migration assay (Figure 2D,E) and CD31^+^ microvessel counts by immunofluorescence (Figure 2F,G), also showed significant elevations in OGD/R‐treated cells compared with the control group. Therefore, the proliferation, migration and proangiogenic capacity of ECs exposed to OGD/R increased.

### 3.3 MALAT1 is an angiogenesis‐associated lncRNA

**Figure 3:**
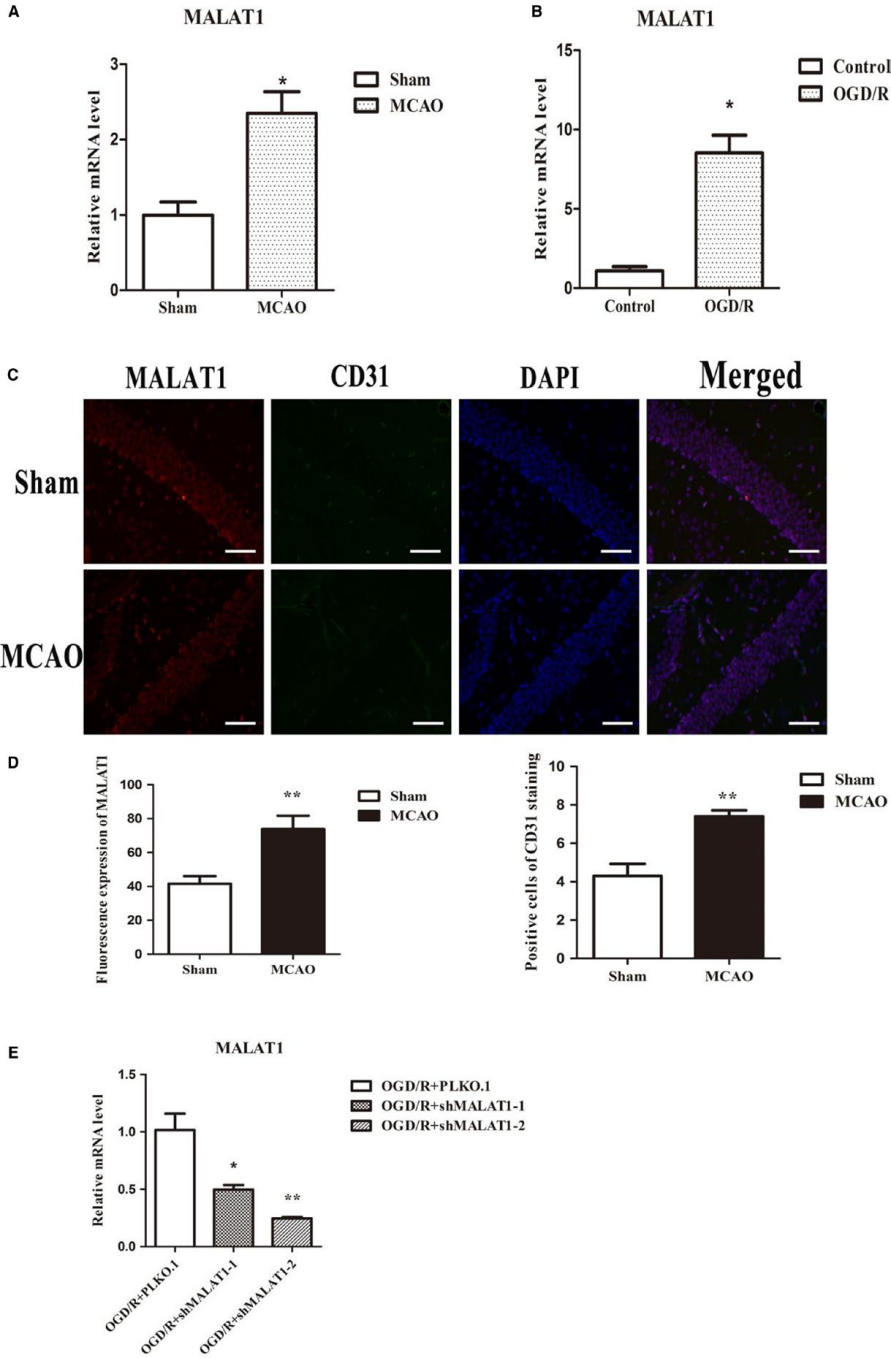
Relative expression of MALAT1. (A, B) The up‐regulation of relative expression of MALAT1 caused by I/R and oxygen‐glucose deprivation/reoxygenation (OGD/R). Data are presented as the mean ± SEM (n = 6/group). *P < 0.05 vs sham and control. (C) MALAT1 and CD31 were detected in mice cerebral tissues of sham group and MCAO group by using fluorescence in situ hybridization, scale bar = 50 μm. (D) Summary graph depicting the expression of MALAT1 and CD31 in sham group and MCAO group. (E) Lentiviral transfection with two different interference sequences targeting MALAT1. Data are presented as the mean ± SEM (n = 6/group). *P < 0.05 vs empty vector, **P < 0.01 vs empty vector

To investigate the role of MALAT1 in the processes of angiogenesis following I/R, total RNA was extracted from the ischaemic penumbra following I/R. Then, we examined MALAT1 expression. We found that MALAT1 expression was aberrantly up‐regulated in I/R (Figure 3A). Moreover, we observed that the expression of MALAT1 in ECs was significantly increased in response to OGD/R in vitro (Figure 3B). Taken together, these results show that ischaemia or hypoxia can induce the up‐regulation of MALAT1 both in vivo and in vitro.

In addition, MALAT1 expression was mostly located in the nuclei of cells according to FISH. The MALAT1 expression level was significantly greater in the MCAO group than that in the sham group (Figure 3C). Moreover, we found that the expression of angiogenesis‐associated marker CD31, which was located in the endothelial cells of microvessels, and showed a trend similar to that of MALAT1 in the sham and MCAO groups (Figure 3C,D). The results of FISH confirmed that MALAT1 expression may affect CD31 expression in murine cerebral tissues. Therefore, the experimental evidence supported our hypothesis and demonstrated that MALAT1 may be involved in ischaemia or hypoxia/reperfusion induced angiogenesis of cerebrovascular endothelial cells.

### 3.4 Knockdown of MALAT1 reduces ECs proliferation and migration

**Figure 4:**
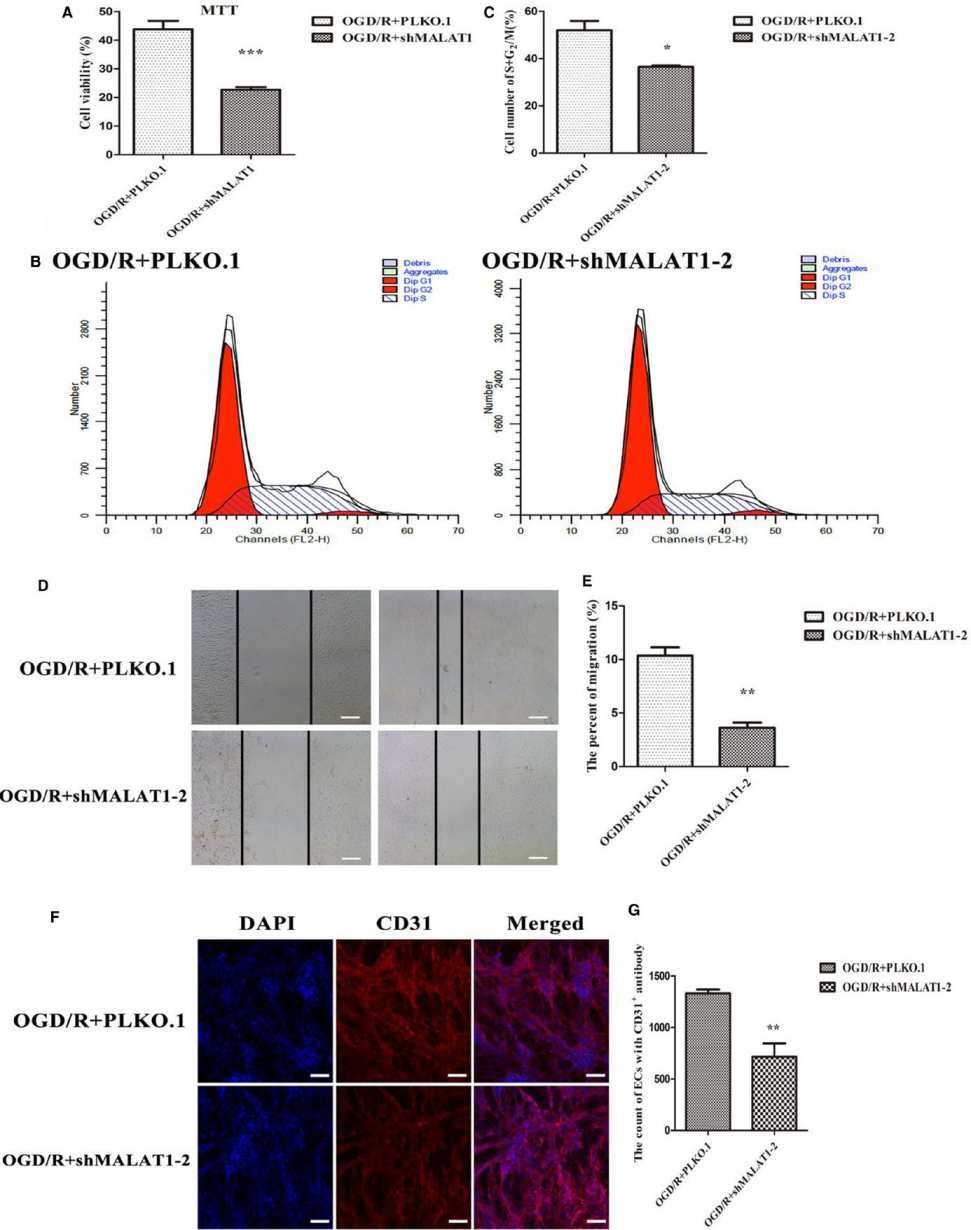
The up‐regulation of endothelial cell survival, proliferation and neovascularization in response to oxygen‐glucose deprivation/reoxygenation (OGD/R) is attenuated by knockdown of MALAT1. (A) Knockdown of MALAT1 reduces the viability of endothelial cells after OGD/R. (B) Knockdown of MALAT1 reduces the proliferation of endothelial cells after OGD/R. (C) Statistical analysis of the number of S + G_2_/M cell after transfection with MALAT1 (n = 6/group). (D) Representative image of migration induced by shMALAT1 and an empty vector. (E) Statistical analysis of migration capacity in cells treated with shMALAT1 compared with an empty vector (n = 6/group). (F) Transfection with MALAT1 prevented elevation of CD31 levels in endothelial cells after OGD/R. (G) Summary graph depicting the CD31 signal intensity, which was significantly decreased after treatment with shMALAT1 compared with an empty vector (n = 6/group). Data are presented as the mean ± SEM. *P < 0.05 vs empty vector, **P < 0.01 vs empty vector, ***P < 0.001 vs empty vector

To assess the potential function of MALAT1 in the biological processes of angiogenesis, we studied the functional significance of MALAT1 alteration in vitro. Brain microvascular endothelial cells were transfected with a lentivirus to cause a marked reduction in the level of MALAT1 (Figure 3E). Although the reduction of MALAT1 expression was significant in both interference fragments, it was more effectively reduced in the second interference fragment (Figure 3E). Accordingly, we used the second fragment in the subsequent assays. We conducted MTT and cell cycle assays, and found that the cell viability (Figure 4A) and proliferation (Figure 4B,C) were markedly repressed in ECs with MALAT1 knockdown compared with empty vector group. Similarly, the scratching assay showed that decreased MALAT1 impaired the migration capacity of ECs (Figure 4D,E). The results demonstrate that MALAT1 can mediate ECs proliferation and contribute to cell migration.

### 3.5 MALAT1 promotes CD 31‐positive endothelial cell expression

To further investigate the effect of MALAT1 on endothelial cells following OGD/R, we employed immunofluorescent staining and found that the knockdown of MALAT1 markedly reduced the number of CD31‐positive ECs compared to the number in the blank control group (Figure 4F,G). These results suggest that MALAT1 can promote the formation of microvessels in response to OGD/R.

### 3.6 15‐LOX1, STAT3 and VEGF may be involved in angiogenesis induced by MALAT1 following OGD/R

**Figure 5:**
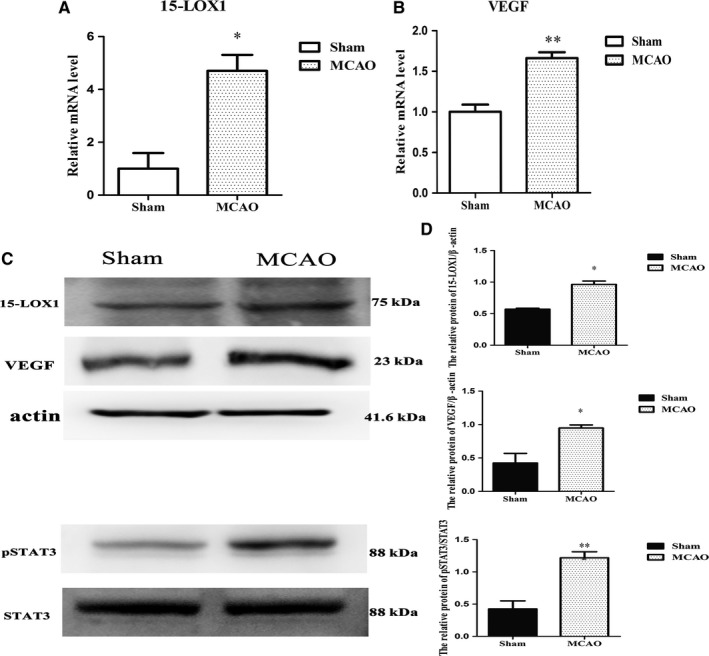
15‐LOX1 and vascular endothelial growth factor (VEGF) up‐regulation at transcription level and the protein level in the MCAO group compared with the sham group. (A) Relative expression level of 15‐LOX1 mRNA. (B) Relative expression level of VEGF mRNA. (C) The protein levels of 15‐LOX1, VEGF and pSTAT3 in the MCAO group compared with the sham group. (D) Statistical analysis of the protein levels of 15‐LOX1, VEGF and pSTAT3 (n = 6/group). Data are presented as the mean ± SEM. *P < 0.05 vs control, ***P < 0.001 vs control,***P <0.001 vs control

**Figure 6:**
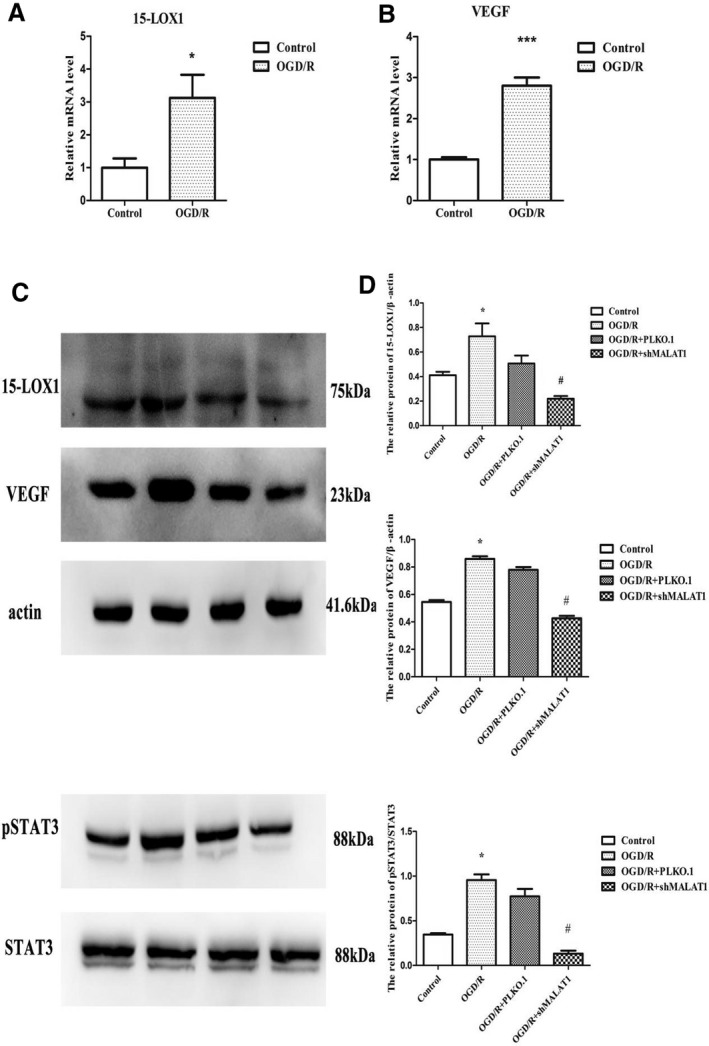
The relative expression of 15‐LOX1 and vascular endothelial growth factor (VEGF) in vitro. (A) The relative mRNA levels of 15‐LOX1 in oxygen‐glucose deprivation/reoxygenation (OGD/R) compared with control. (B) The relative mRNA levels of VEGF in OGD/R compared with control. (C) The protein levels of 15‐LOX1, VEGF and pSTAT3 in cells exposed to control, OGD/R, OGD/R+PLKO.1 and OGD/R+shMALAT1. (D) Statistical analysis of the protein levels of 15‐LOX1, VEGF and pSTAT3 in control, OGD/R, OGD/R+PLKO.1 and OGD/R+shMALAT1 treatment (n = 6/group). Data are presented as the mean ± SEM.*P < 0.05 OGD/R vs control, ** *p* < 0.01 versus OGD/R #*P *< 0.05 OGD/R + shMALAT1 vs OGD/R + PLKO.1. There was no significant difference between OGD/R and OGD/R + PLKO.1

Our results have demonstrated the transcription levels of 15‐LOX1 and VEGF, two important factors in the progression of vascular diseases, were significantly up‐regulated in vivo and in vitro (Figure 5A,B; 6A,B), and 15‐LOX1, VEGF and pSTAT3 were also significantly up‐regulated at the protein level (Figure 5C,D; 6C,D) in vivo and in vitro. However, upon knockdown of MALAT1 compared with PLKO.1, the increases in 15‐LOX1, pSTAT3 and VEGF expression at the protein level were reversed (Figure 6C,D). Therefore, our findings indicate that MALAT1 may not only give rise to angiogenesis in response to ischaemic insults in cerebrovascular endothelial cells, but also promote angiogenesis by regulating the expression of 15‐LOX1, STAT3 and VEGF.

### 3.7 MALAT1 promotes angiogenesis through the activation of the 15‐LOX1/STAT3 signalling pathway

**Figure 7:**
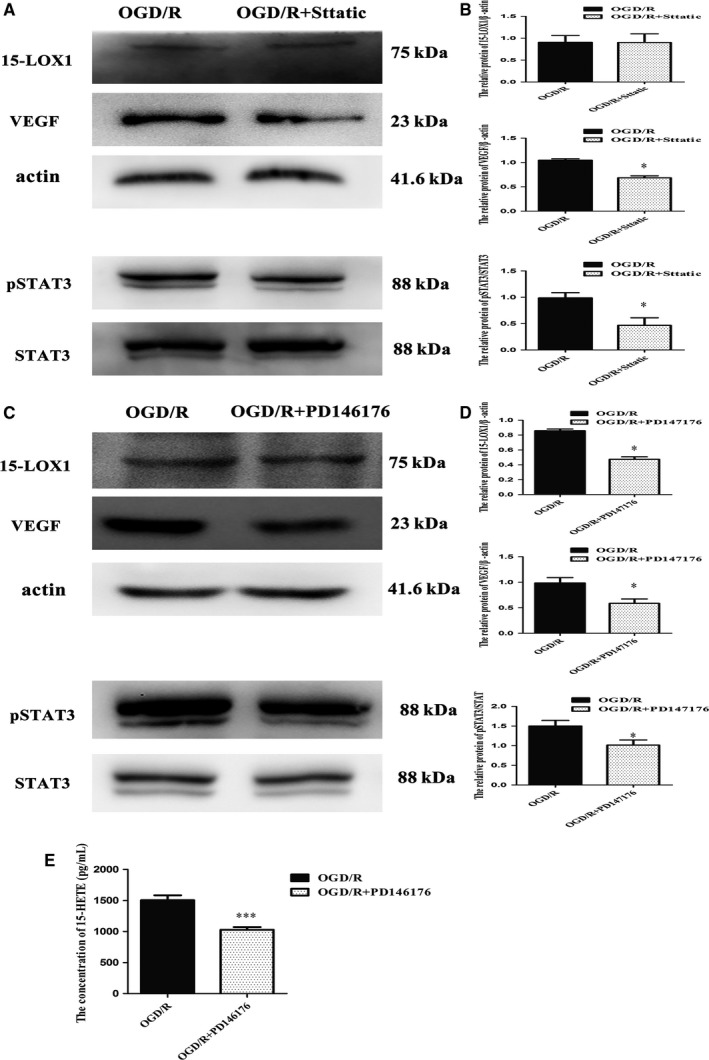
(A, B) The protein levels of 15‐LOX1, vascular endothelial growth factor (VEGF) and pSTAT3 in Stattic‐treated cells compared with control cells. (C, D) The protein levels of 15‐LOX1, VEGF and pSTAT3 in PD146176‐treated cells compared with control. (E) The concentration of 15‐HETE (pg/mL) was determined by enzyme‐linked immunosorbent assay. Data are presented as the mean ± SEM.*P < 0.05 vs oxygen‐glucose deprivation/reoxygenation (OGD/R), **P < 0.01 vs OGD/R, ***P < 0.001 vs OGD/R

Brain microvascular endothelial cells were treated with the small‐molecule STAT3 inhibitor Stattic (MCE, New Jersey, USA) at 2 mg/L. Stattic inhibits the phosphorylation of STAT3,^18^ as detected by a reduction in the phosphorylation of STAT3 at the protein level (Figure 7A,B). Next, we observed the protein expression levels of 15‐LOX1 and VEGF. We found that the exogenous inhibitor of STAT3 could effectively reverse the up‐regulation of VEGF at the protein level (Figure 7A,B), but the expression of 15‐LOX1 was not affected (Figure 7A,B). Stattic treatment distinctly reduced the expression of pSTAT3 and VEGF at the protein level, but those changes were not accompanied by down‐regulation of 15‐LOX1 at the protein level. These data suggest that 15‐LOX1 might be the upstream regulator of STAT3 and VEGF.

### 3.8 STAT3 is a target gene of 15‐LOX1 in angiogenesis following OGD/R

In the last portion of article, we sought to further examine whether 15‐LOX1 is also required for the phosphorylation of STAT3 involved in angiogenesis induced by OGD/R. 6,11‐Dihydro‐5‐thia‐11‐aza‐benzo[a]‐fluorene (PD 146176, Cayman, Ellsworth, USA), as a specific and selective 12/15‐LO competitive inhibitor,^19^, ^20^ can block the conversion of arachidonic acid to 12‐hydroxyeicosatetraenoic acid (12‐HETE) and 15‐LOX1. Therefore, PD146176 can reverse the up‐regulation of 15‐LOX1 (Figure 7C,D). Moreover, the concentration of 15‐HETE which is the metabolites of 12/15‐LO was determined in the samples by comparing the OD values; it is found that PD1461761 can decrease the concentration of 15‐HETE (Figure 7E). We also measured the protein expression levels of pSTAT3 and VEGF in OGD/R ECs treated with PD146176 at 0.75 μmol/L. Likewise, we found that compared with control, PD146176‐treated cells also had decreased levels of pSTAT3 and VEGF (Figure 7C,D). Therefore, we conclude that the activation of STAT3 requires 15‐LOX1 and that STAT3 is a target gene of 15‐LOX1 in angiogenesis induced by OGD/R.

## 4 DISCUSSION

Stroke, as a cerebrovascular accident, causes a loss of brain function due to a disturbance in the blood supply to the brain. Following stroke, the affected area of the brain cannot function normally, which may result in disability and even death.^21^ Substantial efforts are still being devoted to deciphering the complex mechanisms of stroke. Interestingly, accumulated studies have shown that angiogenesis is activated after stroke and that higher neovascular density is associated with less morbidity, disability and mortality.^5^ Therefore, angiogenesis has been recognized as a key to the recovery of brain function.^5^ LncRNAs have been demonstrated to be one of the most abundant classes of ncRNAs.^22^ As the versatile roles of lncRNAs in biological processes and human disorders are increasingly recognized, these RNAs are attracting more extensive attention in the fields of molecular biology and clinic research.^23^ Furthermore, lncRNAs are reported to be potential diagnostic biomarkers and therapeutic targets for multiple diseases^.^ 24 In particular, lncRNAs play a role as a novel type of master regulator after ischaemic stroke. MALAT1 was initially recognized as a tumour‐associated lncRNA‐mediating cancer metastasis and cell survival.^25^, ^26^ Although there is no direct evidence focusing on the involvement of MALAT1 in angiogenesis induced by stroke, MALAT1 has been recognized to be overexpressed in migration, invasion, metastasis and angiogenesis in cancer^27^, ^28^ Moreover, a high expression level of MALAT1 was observed in cultured endothelial cells via in situ hybridization.^29^, ^30^ In the present study, MALAT1 was significantly overexpressed in vivo MCAO and vitro OGD/R compared with sham and control according to the results of the PCR analysis. It was also identified that MALAT1 expression was greater in the MCAO group than in sham group, as revealed by FISH. The expression of CD31, an endothelial cell marker, had the same trend as MALAT1 expression. Therefore, we could conclude that MALAT1 expression may affect the expression of the angiogenesis‐associated marker, CD31 in murine cerebral tissues following I/R. In vitro experiment, the ECs proliferation, migration and the number of CD31 positive cells were used to assess vessel recovery and formation.^31^ Subsequently, we revealed that MALAT1 could promote cerebral ECs proliferation, migration and the number of CD31‐positive cells following OGD/R. In contrast, the knockdown of MALAT1 in cerebrovascular endothelial cells could decrease cell proliferation, migration and the number of CD31 positive cells. Our findings suggest that MALAT1 acts as an angiogenesis‐associated lncRNA that plays a potentially protective role against ischaemic insults. Therefore, in light of the significance of MALAT1 in angiogenesis, it may be a novel therapeutic target for hypoxic injury. Moreover, our findings regarding mechanisms through which MALAT1 modulates angiogenesis will also have profound therapeutic implication, although further research is still needed.

Lipoxygenases (LOXs) are non‐haeme iron dioxygenases that stereospecifically introduce molecular oxygen into polyunsaturated fatty acids, resulting in the formation of hydroperoxyeicosatetraenoic acids, which are subsequently converted to HETEs.^32^ Two 15‐LOXs, including 15‐LOX1 and 15‐LOX2, have been shown to be widely expressed.^33^ Additionally, 15‐LOX1 has been observed as an important factor in the pathogenesis of atherosclerosis and pulmonary artery hypertension, and up‐regulated in pulmonary artery adventitial fibroblasts under hypoxia^.^ 31 Accumulating evidence has determined that 15‐LOX1 plays a critical role in vascular diseases,^34^ including being aberrantly expressed in transient focal ischaemic stroke and mediating superoxide production in vascular smooth muscle cells (VSMCs).^35^, ^36^ Although the expression and function of 15‐LOX1 are well defined in the central nervous system, few studies have clarified the functional mechanisms of 15‐LOX1 in angiogenesis in response to I/R.

STAT3 has also been demonstrated to be an important player in cellular processes including proliferation and migration.^19^, ^25^ Many studies have reported that STAT3 is activated and translocated into the nucleus upon exposure to hypoxia. Its increased expression and activation may be linked to cardiovascular diseases, such as atherosclerosis and stroke. STAT3 has also been demonstrated to mediate VSMC growth and migration in response to ischaemic injury^37^, ^38^ Moreover, STAT3‐mediated cerebral ischaemic tolerance has been well characterized and can affect the brain parenchyma and vasculature associated cells, providing not only neuroprotection but also cerebral blood flow in the presence of pathology. The knockdown of STAT3 increases the injury to the brain.^39^ In this study, analysis of 15‐LOX1 and STAT3 expression has led to the detection of abundant 15‐LOX1 and STAT3 in I/R and OGD/R in accordance with MALAT1 expression. In contrast, the overexpression of 15‐LOX1 and STAT3 was abolished by knockdown of MALAT1, indicating that MALAT1 promotes angiogenesis by regulating the expression of 15‐LOX1 and STAT3.

Vascular endothelial growth factor is the best candidate regulator of endothelial cell growth and differentiation among the factors capable of modulating angiogenesis. Increasing evidence shows that the VEGF‐induced the physiological processes of angiogenesis after ischaemia are regulated by STAT3. Conversely, transfection with STAT3 almost completely inhibited VEGF‐induced EC migration and tube formation.^40^ In our studies, analysis of 15‐LOX1, STAT3 and VEGF expression has led to the detection of abundant 15‐LOX1, STAT3 and VEGF in angiogenesis after I/R and OGD/R in accordance with MALAT1 expression. In contrast, the up‐regulation of 15‐LOX1, STAT3 and VEGF was alleviated by the knockdown of MALAT1. Therefore, we report for the first time that MALAT1 promotes angiogenesis through regulating the expression of 15‐LOX1, STAT3 and VEGF. However, which gene is the direct target of MALAT1 remains to be explored. Regarding proangiogenic effects, previous studies showed a close interaction between 15‐LOX1 and STAT3, in which 15‐LOX1 production stimulated STAT3 tyrosine phosphorylation in both VSMCs and pulmonary artery endothelial cells^33^, ^41^, ^42^ Adenovirus‐mediated down‐regulation of 15‐LOX1 suppressed the injury‐induced phosphorylation of STAT3.^40^ However, the interaction of 15‐LOX1 and STAT3 in response to the complex mechanisms of angiogenesis mediated by MALAT1 after OGD/R is not yet well understood. In our study, Stattic was used to inhibit the phosphorylation of STAT3; furthermore, inhibitor of STAT3 tyrosine phosphorylation alleviated the increase in VEGF, but the expression of 15‐LOX1 did not change. PD146176, an inhibitor of 15‐LOX1, was also shown to abolish the up‐regulation of pSTAT3 and VEGF in the present study. Based on our observation, it is conceivable that 15‐LOX1 is an upstream factor of STAT3 and can regulate its phosphorylation. Herein, we elaborated that STAT3 and VEGF, as downstream target genes of 15‐LOX1, participate in angiogenesis mediated by MALAT1 under hypoxic conditions. The inhibitors of 15‐LOX1 and pSTAT3 can reduce the proangiogenic function of MALAT1 by reducing VEGF expression. However, recent evidence has demonstrated that lncRNAs can serve as sponges to titrate microRNAs (miRNAs) and prevent them from binding to mRNAs by acting as competing endogenous RNAs (ceRNAs).^44^ In the development of non‐small cell lung cancer (NSCLC), previous studies have uncovered that MALAT1 can act as a ceRNA to modulate miR124/STAT3.^43^ Therefore, it is unclear whether there is a negative correlation between MALAT1 and some miRNAs in angiogenesis following I/R. Additionally, other potential targets of MALAT1 and the functional roles of some miRNAs between MALAT1 and 15‐LOX1 or STAT3 were not investigated in this article and still need to be further studied.

## 5 CONCLUSIONS

In summary, our findings are the first to demonstrate that the angiogenesis‐associated lncRNA MALAT1 exerts proangiogenic effects through the 15‐LOX1/STAT3 signalling pathway in I/R and OGD/R, which provides a detailed understanding of angiogenesis after OGD/R injury. Therefore, MALAT1 expression is augmented in brain endothelial cells under ischaemia, which may contribute to a potential therapeutic strategy for patients who have suffered ischaemic stroke.

## DISCLOSURE

There is no conflict of interests.
